# Escort cell encapsulation of *Drosophila* germline cells is maintained by irre cell recognition module proteins

**DOI:** 10.1242/bio.039842

**Published:** 2019-03-15

**Authors:** Doreen S. Ben-Zvi, Talila Volk

**Affiliations:** Department of Molecular Genetics, Weizmann Institute of Science, Rehovot 76100, Israel

**Keywords:** Germline stem cells, Escort cells, *Drosophila*, Egfr, Germarium

## Abstract

Differentiation of germline stem cells (GSCs) in the *Drosophila* ovary is induced by somatic escort cells (ECs), which extend membrane protrusions encapsulating the germline cells (GCs). Germline encapsulation requires activated epidermal growth factor receptor (Egfr) signaling within the ECs, following secretion of its ligands from the GCs. We show that the conserved family of irre cell recognition module (IRM) proteins is essential for GC encapsulation by ECs, with a requirement for *roughest* (*rst*) and *kin of irre* (*kirre*) in the germline and for *sticks and stones* (*sns*) and *hibris* (*hbs*) in ECs. In the absence of IRM components in their respective cell types, EC extensions are reduced concomitantly with a decrease in Egfr signaling in these cells. Reintroducing either activated Egfr in the ECs, or overexpressing its ligand Spitz (Spi) from the germline, rescued the requirement for IRM proteins in both cell types. These experiments introduce novel essential components, the IRM proteins, into the process of inductive interactions between GCs and ECs, and imply that IRM-mediated activity is required upstream of the Egfr signaling.

## Abstract (precis)

**Statement:** IRM proteins mediate adhesion between escort cells and the germline cells, promoting short range signaling required for escort cells’ encapsulation of germline cells.

## INTRODUCTION

Stem cell function depends on proper input from their environment. Anchoring of the germline stem cells (GSCs) to the maintenance niche is essential for them to preserve a stem cell state (Song and Xie, 2002; Xie and Spradling, 2000), and the encapsulation of the differentiating germline cells (GCs) by escort cells (ECs) is a prerequisite for their differentiation (Kirilly et al., 2011; Maimon et al., 2014; Su et al., 2018). While the anchoring process and its contribution to GSCs maintenance have been well studied, much less is known about the nature of the encapsulation process and its contribution to GCs differentiation.

Each adult *Drosophila* ovary is composed of 16–20 ovarioles, which serve as egg production lines. Continuous egg production depends on GSCs, which are housed at the anterior tip of each ovariole, in the germarium (Kirilly and Xie, 2007; Xie and Spradling, 2000). The cap cells of the maintenance niche and the GSCs express the DE-cadherin *shotgun*, which mediates their attachment to each other, and lack of DE-cadherin results in stem cell loss (Song and Xie, 2002). Close proximity between the stem cells and the maintenance niche is vital for the GSCs to receive the BMP2/4 homologue *decapentaplegic* (Dpp) from the cap cells (Kai and Spradling, 2003; Xie and Spradling, 2000)*.* Dpp signaling in the GSCs results in phosphorylation of Mad (Mothers against Dpp, a SMAD2/3 homologue) and repression of *bam* (*bag-of-marble*s) expression, a key germline differentiation effector (Chen and McKearin, 2003; Ohlstein and McKearin, 1997; Song et al., 2004).

The GSCs undergo asymmetric division, with one daughter cell remaining in the niche and replenishing the stem cell reservoir. The other daughter cell, the cystoblast, exits the maintenance niche and begins to differentiate (Xie and Spradling, 2000). During differentiation, GCs undergo incomplete cell divisions and remain connected by cytoplasmic bridges, thereby forming cysts (de Cuevas et al., 1997). The fusome, an intracellular organelle connecting GCs, changes shape throughout GCs’ differentiation from a round structure in GSCs and in cystoblasts, into a branched profile in differentiated cysts (de Cuevas et al., 1997; Lin et al., 1994).

Exiting the maintenance niche is a necessary but not sufficient step towards GSC differentiation, and the latter is further supported by ECs, which provide essential signals for differentiation. ECs encapsulate the GCs with membrane extensions, shown to promote GC differentiation. In the absence of these protrusions, differentiation does not progress (Kirilly et al., 2011; Maimon et al., 2014; Su et al., 2018). JAK/STAT (Maimon et al., 2014; Rozario and DeSimone, 2010) and Egfr (Gilboa and Lehmann, 2006; Liu et al., 2010; Schulz et al., 2002) activation are both required to induce the ECs’ specialized membrane processes (Liu et al., 2010; Maimon et al., 2014; Schulz et al., 2002). The germline supports the EC extensions through the secretion of Egfr ligands (Liu et al., 2010), which in turn activates the Egfr signaling in the ECs (Gilboa and Lehmann, 2006; Liu et al., 2010; Schulz et al., 2002). Recent studies demonstrated that EC membrane extensions behave dynamically, with a subset of them continuously retracting and extending, whereas others become stabilized, allowing firm encapsulation of the germline (Banisch et al., 2017). Such dynamic behavior might provide essential signals for GC differentiation, but still allows physical progression of the differentiated GCs posteriorly. Although some of the factors that act from the soma are known, little is known about the role of GCs in the encapsulation.

In an attempt to identify novel components mediating the cross talk between GCs and ECs, we performed an RNAi-based screen with a group of candidate genes. One hit from this screen was Kirre, a member of the irre cell recognition module (IRM) family. IRM proteins belong to the Ig superfamily of proteins, with conserved structure and function from *C. elegans* and flies, to humans (Fischbach et al., 2009). *Drosophila* IRM family includes four membrane proteins: Rst, Kirre, SNS and Hbs, which are expressed by multiple cell types, where they mediate various heterotypic adhesion processes. These include myoblast fusion to myotubes (Artero et al., 2001; Dworak et al., 2001; Paululat et al., 1995; Ruiz-Gómez et al., 2000; Strunkelnberg et al., 2001), cell sorting during ommatidia formation (Bao and Cagan, 2005; Bao et al., 2010), cell spacing in the olfactory sensory organs, the formation of the wing margins (Venugopala Reddy et al., 1999), axon pathfinding in the optic lobe (Boschert et al., 1990; Schneider et al., 1995), programmed cell death in the eye (Reiter et al., 1996; Wolff and Ready, 1991) and the establishment of a slit diaphragm-like structure in garland and in pericardial nephrocytes (Weavers et al., 2009; Zhuang et al., 2009). IRM proteins also function in the peritoneal and epithelial muscle sheaths of larval ovaries during egg chamber oogenesis (Valer et al., 2018).

The IRM-mediated attachment is characterized by heterotypic interactions between two paralog sets; Rst and Kirre bind with either Sns or Hbs. In some systems one binding pair is dominant while other systems utilize them redundantly. Additionally, the expression pattern of the different IRM components is not necessarily limited to specific cell types involved in the interaction. In eye development the only active IRM proteins are Rst and Hibris (Bao and Cagan, 2005), with interommatidial precursor cells expressing Rst and primary pigment cells expressing Hibris (Bao and Cagan, 2005). In myoblast fusion, Kirre acts redundantly with Rst (Ruiz-Gómez et al., 2000; Strunkelnberg et al., 2001) and Hbs can partially substitute for Sns (Shelton et al., 2009), with expression of Rst on both fusion competent myoblasts and founder cells (Galletta et al., 2004; Strunkelnberg et al., 2001). During myoblast fusion, IRM proteins promote attachment between the myotube and the myoblast through direct binding of their extracellular domains (Galletta et al., 2004). Subsequently, the cytoplasmic domains of both Kirre and SNS recruit the actin polymerization machinery through verprolin/WASP interacting protein (WIP), a process suggested to support the membrane fusion process (Kaipa et al., 2013; Kim et al., 2007; Massarwa et al., 2007; Mukherjee et al., 2011).

The mammalian homologs of *rst* and of *kirre* are *KIRREL* (*Neph1*), *KIRREL2* (*Neph3*) and *KIRREL*3 (*Neph2*, *mkirre*), while both *sns* and *hbs* have one known homolog, *Nephrin* (Fischbach et al., 2009). These are required for processes that are akin to the *Drosophila* ones: muscle fusion (Sohn et al., 2009; Wagner et al., 2011), axon pathfinding and synapse formation of proprioceptive neurons on muscle spindles (Komori et al., 2008), sensory organ formation (Morikawa et al., 2007), and the establishment of filtration barrier of the kidney podocytes (Tryggvason et al., 2006). In humans, mutations in *Neph1* or *Nephrin* associate with congenital nephrotic syndrome of the Finnish type (NPHS1), in which the glomerular filtration barrier breaks down (Kestilä et al., 1998).

Here we show that the IRM proteins are required for the formation of EC membrane extensions which encapsulate the GCs, as well as for activation of the Egfr signaling pathway within the ECs. Moreover, we show that hyper activation of the Egfr signaling in the ECs bypasses the requirement for IRM proteins-dependent adhesion, supporting a model in which IRM-dependent adhesion acts upstream of the Egfr signaling, to stabilize ECs membrane extensions.

## RESULTS

### Kirre and Rst are required within germline cells for their encapsulation by ECs

In order to identify novel components required for germline encapsulation by ECs, an RNAi-based screen with candidate genes coding for putative membrane or ECM proteins was performed. Two drivers were used, *nanos-*Gal4 (nos-Gal4) driving expression in the germline, and *traffic jam-*Gal4 (*tj*-Gal4), driving expression in the somatic ECs (Bolívar et al., 2006; Li et al., 2003; Van Doren et al., 1998). Encapsulation defects manifest in aberrant ECs extensions, or in abnormal GC differentiation. The extensions were visualized by anti-Coracle (Cora), a membrane protein that is highly expressed in ECs membrane protrusions (Fairchild et al., 2015; Maimon et al., 2014). Differentiation was detected by round fusome amount, visualized by anti-hu-li tai shao (Hts). Summary of the genes identified in this screen is described in Table S1.

**Fig. 1.**
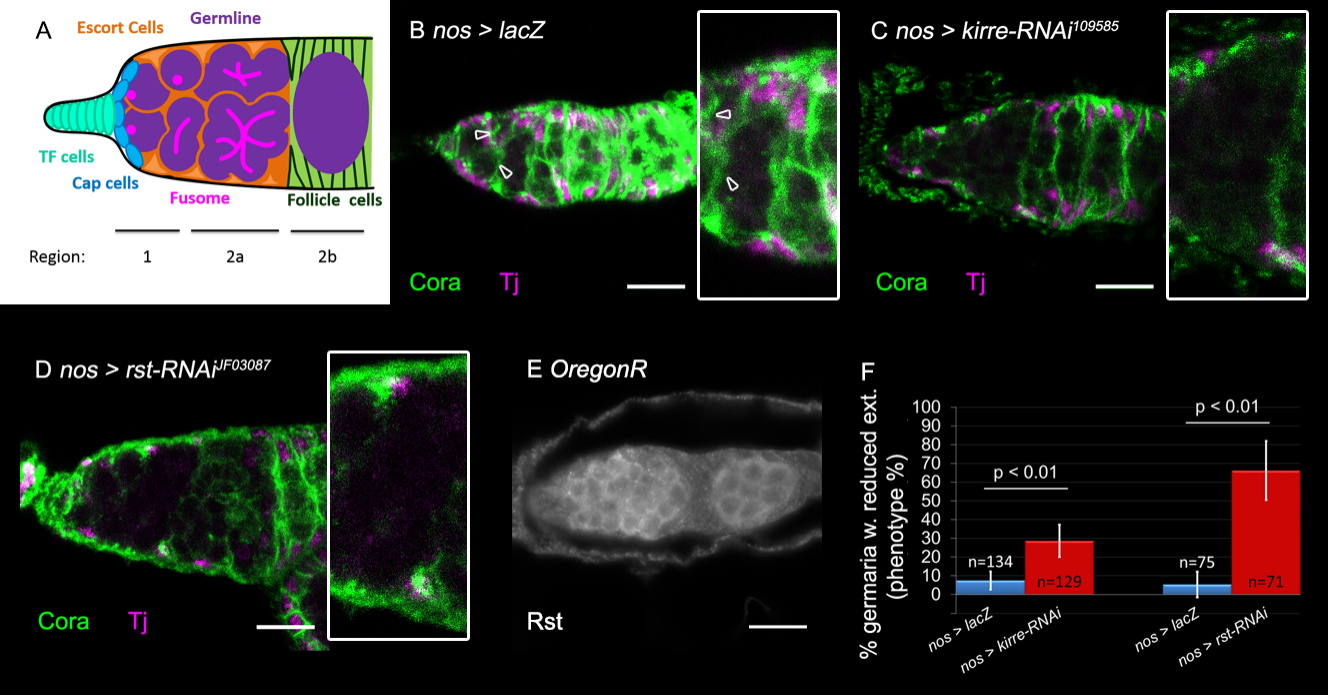
**Germline knockdown of *kirre* or *rst* inhibits ECs membrane extensions.** (A) Schematic representation of the germarium. In region 1 GSCs are attached to the maintenance niche and differentiation begins with their cystoblast daughter cells, all of which contain round fusomes. In region 2a the differentiating germline cells, characterized by branched fusomes, are encapsulated by EC extensions. In region 2b the 16-cell cyst is surrounded by follicle cells. (B–D) Anti-Coracle (Cora, green) marks somatic cell membranes, Anti-Tj marks ECs (magenta) arrowheads point to existing extensions, insets show region 2a magnified twofold. (B) Control germline *nos*-GAL4>*lacZ* germaria exhibits normal EC extensions (green). (C) Representative image of germline knockdown of *kirre* (using *kirre* RNAi) which reduces EC extensions. (D) Representative image of germline knockdown of *rst* (using *rst* RNAi) which reduces EC extensions. (E) Rst staining is present in the germline, as revealed by Anti-Rst. (F) Quantification of the percentage of germaria with reduced EC extensions in germline knockdown of *kirre* (*n*=129, eight independent experiments) or *rst* (*n*=71, six independent experiments). *P*-values were calculated using binomial proportions z-test, bars represent s.d. Scale bars: 10 µm.

An interesting hit of this screen was *kirre*, coding for one of four IRM family members in the *Drosophila* genome. *kirre* knockdown in the germline resulted in a non-autonomous effect on the ECs. In region 2a of the germarium the differentiating germline cells associates with ECs (Fig. 1A). When *kirre* was knocked-down using *kirre-RNAi^109585^*, EC membrane extensions were reduced in this region (Fig. 1, compare B to C). Quantification of this phenotype was achieved by scoring germaria into one of two groups: those lacking extensions in region 2a in at least three consecutive focal planes (z=6 µm) and those with normal extensions in this region in all focal planes. *kirre* knockdown in the germline resulted in 29% germaria with a decreased number of extensions (germaria, *n*=129 *P*<0.01) (Fig. 1F). Notably, this reduction was not due to an absence of ECs in the affected area, as they were still visible when labeled with anti-Traffic jam (Tj) (Fig. 1C). Knockdown of *kirre* using a second RNAi line (*kirre-*RNAi^27227^) caused partial lethality of the animals, and hence was not quantified.

Similarly to *kirre*, *roughest* (*rst*) knockdown in the germline led to a reduction in EC extensions (Fig. 1, compare B to D). Decreased extensions were detected in 66% of analyzed germaria (*n*=71 *P*<0.01) (Fig. 1F), whereas no effect on the presence of the ECs was observed (Fig. 1D). Anti-Rst staining revealed Rst localization along the GC boundaries (Fig. 1E). It was therefore concluded that Kirre and Rst are both required in the germline for a non-autonomous induction of EC membrane extensions.

Knockdown of *kirre* and *rst* in the ECs using *tj*-Gal4, did not lead to a visible phenotype (Fig. S1A-C, quantification in G), confirming the non-autonomous activity of both Kirre and Rst in the GCs. Taken together, these experiments support a non-autonomous function of Kirre and Rst in the germline, essential for inducing membrane extensions of the ECs.

### Hbs and Sns are required in ECs for germ cell encapsulation

**Fig. 2.**
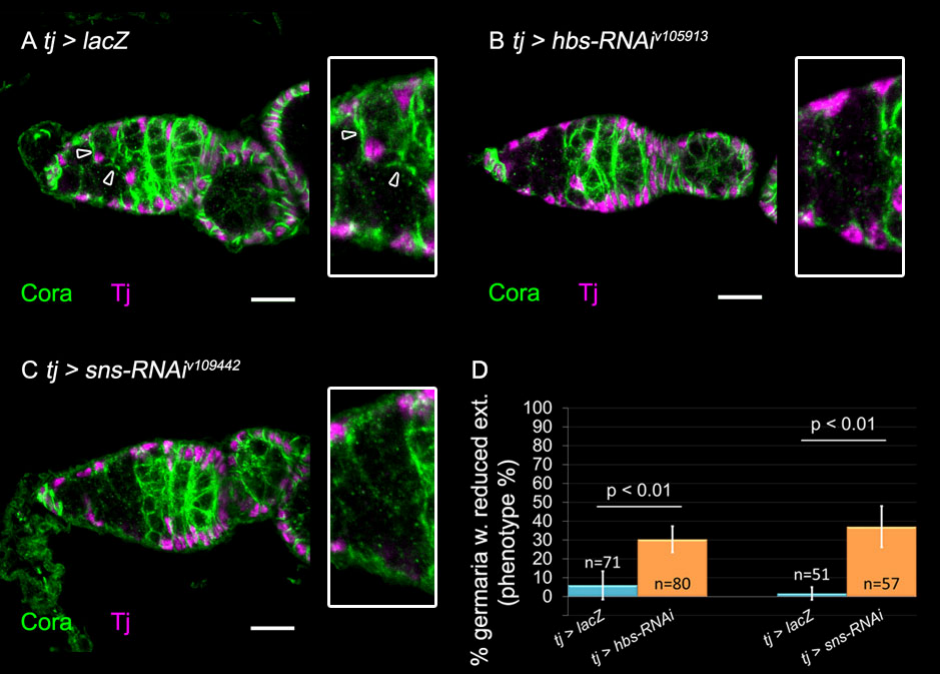
**Somatic knockdown of *hbs* or *sns* inhibits EC membrane extension.** In all panels: Anti-Coracle (Cora, green) marks somatic cell membranes, Anti-Tj marks ECs (magenta). Arrowheads point to existing extensions, insets show region 2a magnified twofold. (A) Control *tj*-GAL4>*lacZ* exhibits normal EC extensions. (B) Somatic knockdown of *hbs* using RNAi exhibits reduced EC extensions. (C) Somatic knockdown of *sns* using RNAi exhibits reduced EC extensions. (D) Quantification of the percentage of germaria with reduced extensions observed in somatic knockdown of *hbs* (*n*=80, six independent experiments) or *sns* (*n*=57, four independent experiments). *P*-values were calculated using binomial proportions z-test, bars represent s.d. Scale bars: 10 µm.

Next, we knocked down *hbs* and *sns*, the binding partners of *kirre* and *rst*, either in the germline or in the soma, driving their corresponding RNAi lines with either soma (*tj*) or germline (*nos*) GAL4 drivers. *hbs* knockdown in the soma resulted in 30% of analyzed germaria presenting reduced extension in region 2a (*n*=80 *P*<0.01) (Fig. 2, compare A to B, quantification in D), without affecting EC number. Similarly, knockdown of *sns* in the ECs led to a phenotype of reduced EC membrane extensions in region 2a in 37% of the analyzed germaria (*n*=57 *P*<0.01) (Fig. 2, compare A to C, quantification in D), with no effect on EC number. In contrast, knockdown of *hbs* or *sns* in the germline did not impact the formation of EC extensions (Fig. S1D-F, quantification in H). It was therefore concluded that whereas Kirre and Rst promote EC extensions non-autonomously acting within the GCs, Sns and Hbs activities are required autonomously in the ECs.

### The requirement for IRM components is haploinsufficient

**Fig. 3.**
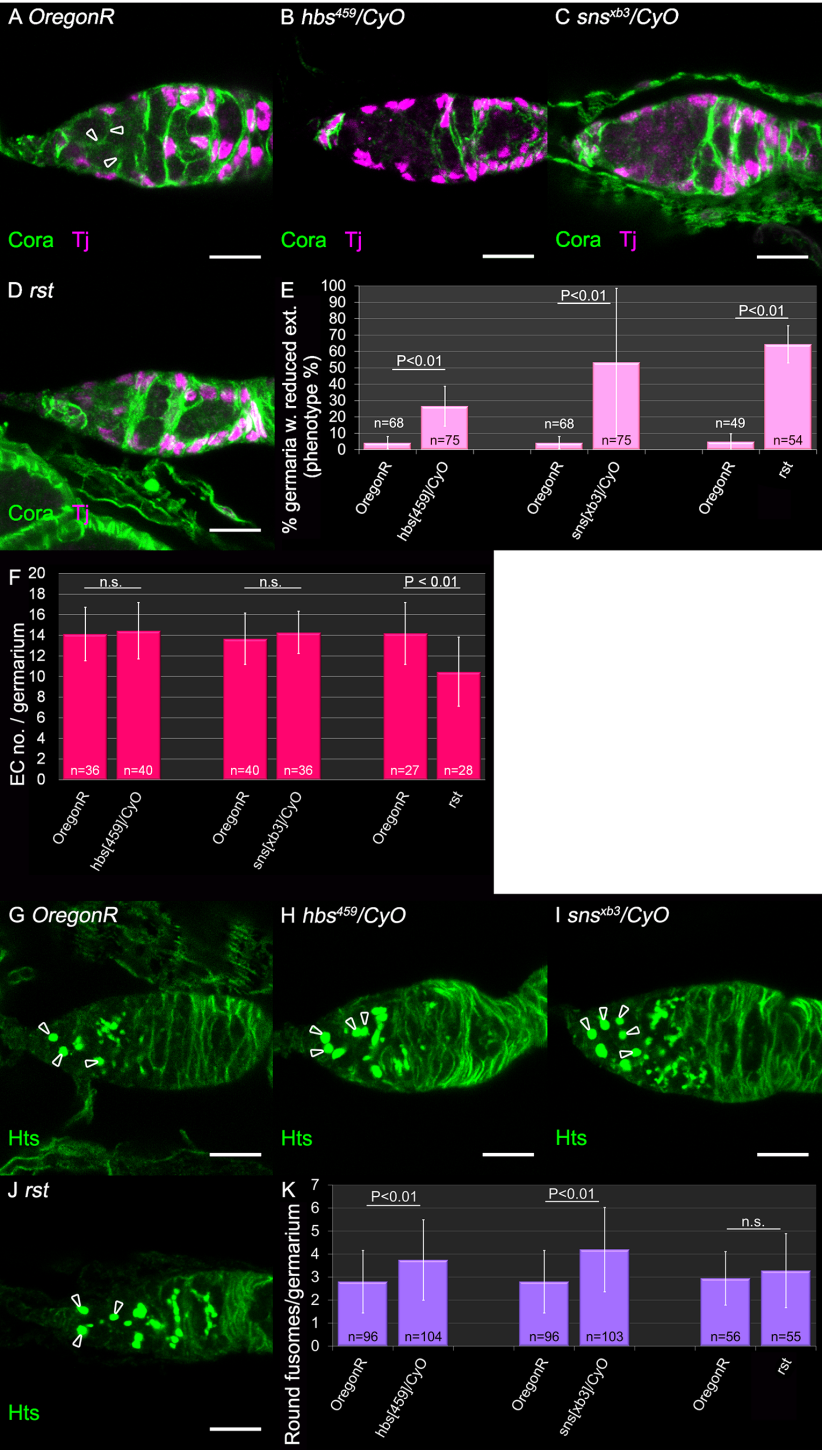
**Haplo insufficient requirements for IRM genes in inducing EC membrane extensions and in differentiation.** (A) WT germarium, each germline cell is surrounded by EC extensions (arrowheads). Anti-Coracle (Cora, green) marks somatic cell membranes, Anti-Tj marks ECs (magenta). (B,C) Representative images of heterozygous mutants of *hbs* (B) or *sns* (C) exhibit reduced extensions. (D) Homozygous *rst* mutant has fewer extensions. (E) Quantification of the percentage of germaria with reduced extensions in heterozygous females of *hbs* (*n*=75)*, sns* (*n*=75)*, rst* (*n*=54) mutants. For each mutation, five independent experiments are shown. *P*-values were calculated using binomial proportions z-test, bars represent s.d. (F) Quantification of ECs present in heterozygous germaria of *hbs* (*n*=40, three independent experiments), *sns* (*n*=36, three independent experiments), *rst* (*n*=28, two independent experiments) mutants. *P*-values were calculated using two-sample *t*-test, bars represent s.d. (G) WT germline contains about three germline cells with round fusomes (arrowheads). Anti-Hts (green) stains fusomes and membranes. (H,I) Representative heterozygous mutant germarium of *hbs* (H) or *sns* (I) contain more GCs with round fusomes, indicating undifferentiated germlines (arrowheads). (J) Homozygous *rst* mutant germarium contains about three round fusomes (arrowheads). (K) Quantification of the number of round fusomes per germarium in heterozygous females of *hbs* (*n*=104, seven independent experiments), *sns* (*n*=103, seven independent experiments), and *rst* (*n*=55, four independent experiments). *P*-values were calculated using two-sample *t*-test, bars represent s.d. Scale bars: 10 µm.

Homozygous null mutant embryos for each of the IRM genes do not survive to adulthood due to their requirements in other tissues. Interestingly, ovaries heterozygous for either *hbs^459^*, sns^xb3^, as well as homozygous *rst* hypomorphic allele (rst^MI04842-GFSTF1^) did show a phenotype of loss of ECs extensions (Fig. 3A–D, and quantification in E). A significant reduction of EC membrane extensions was observed in 27% of *hbs^459^/CyO* germaria (*n*=75 *P*<0.01) (Fig. 3B,E)*,* as well as in 53% germaria of *sns^xb3^/CyO* (*n*=75 *P*<0.01) (Fig. 3C,E) and in 42% of *sns^s660^/CyO* germaria (*n*=36 *P*<0.01) (Fig. S2A,B). Germaria of *rst* homozygous hypomorphic allele had fewer extensions in 64% of the germaria (*n*=54 *P*<0.01) (Fig. 3D,E). The number of ECs remained constant in all of the heterozygous background, while *rst* homozygous hypomorphic germaria had less ECs and were smaller (*n*=28 *P*<0.01) (Fig. 3F, Fig. S2C). Interestingly, *kirre,rst* heterozygous females carrying a deficiency that deletes both genes did not exhibit a phenotype of lack of ECs extensions, possibly pointing to an unequal functional contribution of the IRM proteins to GC–EC adhesion (Fig. S3).

These results corroborate the conclusions obtained by the RNAi experiments, and support the direct involvement of the IRM proteins in mediating communication between the germline and ECs, required for germline encapsulation by the ECs. We also produced mutant clones for the IRM alleles in the soma or in the germline, to evaluate the phenotype of EC extensions in a complete absence of either of the IRM components. Unfortunately, EC mutant clones were less frequent then control EC clones and were often comprised of a single cell, making it difficult to distinguish between its own extensions and extensions from neighboring wild-type (WT) EC. Germline clones were of equal frequency as in the control. However, completely mutant germline was not achieved, so that each EC always came into contact with WT germline and we were unable to infer the outcome of full absence of the relevant IRM (Fig. S4 shows that the mutant cell is still in contact with one WT cell).

Encapsulation of GCs by ECs had been shown previously to contribute to differentiation of the GCs, detected by a transformation of the round fusomes into elongated ones (Kirilly et al., 2011; Lim and Fuller, 2012; Maimon et al., 2014; Schulz et al., 2002; Su et al., 2018). We further analyzed the degree of GCs differentiation by quantifying the number of round fusomes (not including dividing GSCs) in the germaria of either *hbs*, or *sns* heterozygous, as well as in *rst* hypomorphic homozygous females, all of which exhibited a phenotype of loss of EC membrane extensions. *hbs^459^/CyO* germaria did exhibit higher number of rounded fusomes (*n*=104 *P*<0.01) (Fig. 3H, quantification in K). Similarly, *sns^xb3^/CyO* germaria were found to contain a higher number of rounded fusomes (*n*=103 *P*<0.01) (Fig. 3I, quantification in K). However, heterozygotes *sns^s660^/CyO* (Fig. S2D-E), or *rst* hypomorphic homozygous (Fig. 3J, quantification in K) did not exhibit a significant increase in the number of round fusomes. These results support the notion that a primary role of IRM proteins is mediating EC extensions rather than directly affecting differentiation of the GCs. RNAi mediated knockdown for *rst*, *hbs*, *sns*, but not for *kirre* similarly exhibited increased number of round fusomes (Fig. S5A,B).

### IRM proteins are required for Egfr signaling in ECs

**Fig. 4.**
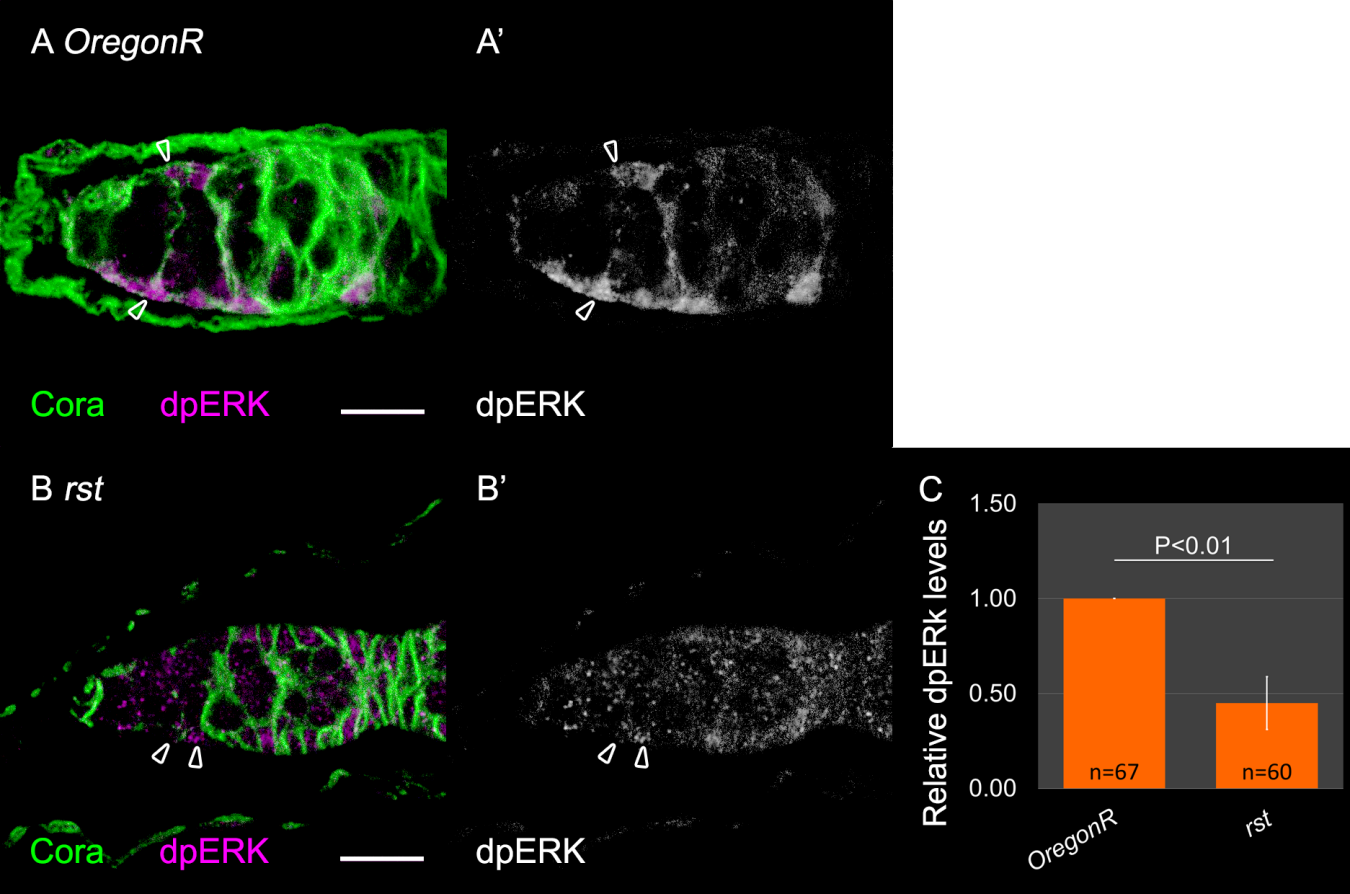
***rst* is required for Egfr** **activation in ECs.** In all panels: Anti-Coracle (Cora, green) marks somatic cell membranes, Anti-dpERK (magenta) marks somatic cell bodies. (A,A′) Control ECs express dpERK. (B,B′) *rst* homozygous mutant ECs have reduced dpERK levels. Arrowheads indicate ECs. (C) Quantification of dpERK fluorescent levels in ECs of control (OregonR, *n*=67) or *rst* (*n*=60) mutant germaria. Six independent experiments are shown. *P*-values were calculated using two-sample *t*-test, bars represent s.d. Scale bars: 10 µm.

Two signaling pathways, Egfr, as well as JAK/STAT had been previously shown to promote the formation of EC membrane extensions. To assess whether activation of these pathways is downstream of IRM-dependent communication between ECs and GCs, we compared the levels of their respective targets in *rst* homozygous mutant germaria. Namely, double phosphorylated ERK (dpERK) for Egfr (Kirilly et al., 2011; Schulz et al., 2002), and the transcriptional target ZFh1 for JAK/STAT (Leatherman and Dinardo, 2010; Maimon et al., 2014; Singh et al., 2010). Zfh1 levels in the ECs were unaffected in *rst* homozygous mutant germaria (Fig. S6), suggesting no connection between IRM and JAK/STAT signaling. In contrast, dpERK levels were significantly reduced in *rst* homozygous mutant ECs (fluorescence quantification indicated that the intensity of dpERK was 0.45-fold less than that of control, *n*=60 *P*<0.01) (Fig. 4), suggesting that IRM-dependent EC–GC adhesion is upstream of Egfr activation in the ECs. Analysis of heterozygous mutant germaria did not show a statistically significant change in dpERK signaling, unlike the analysis of the *rst* homozygous mutant germaria, possibly indicating a different sensitivity of the dpERK intensity relative to the phenotype of EC membrane extension.

**Fig. 5.**
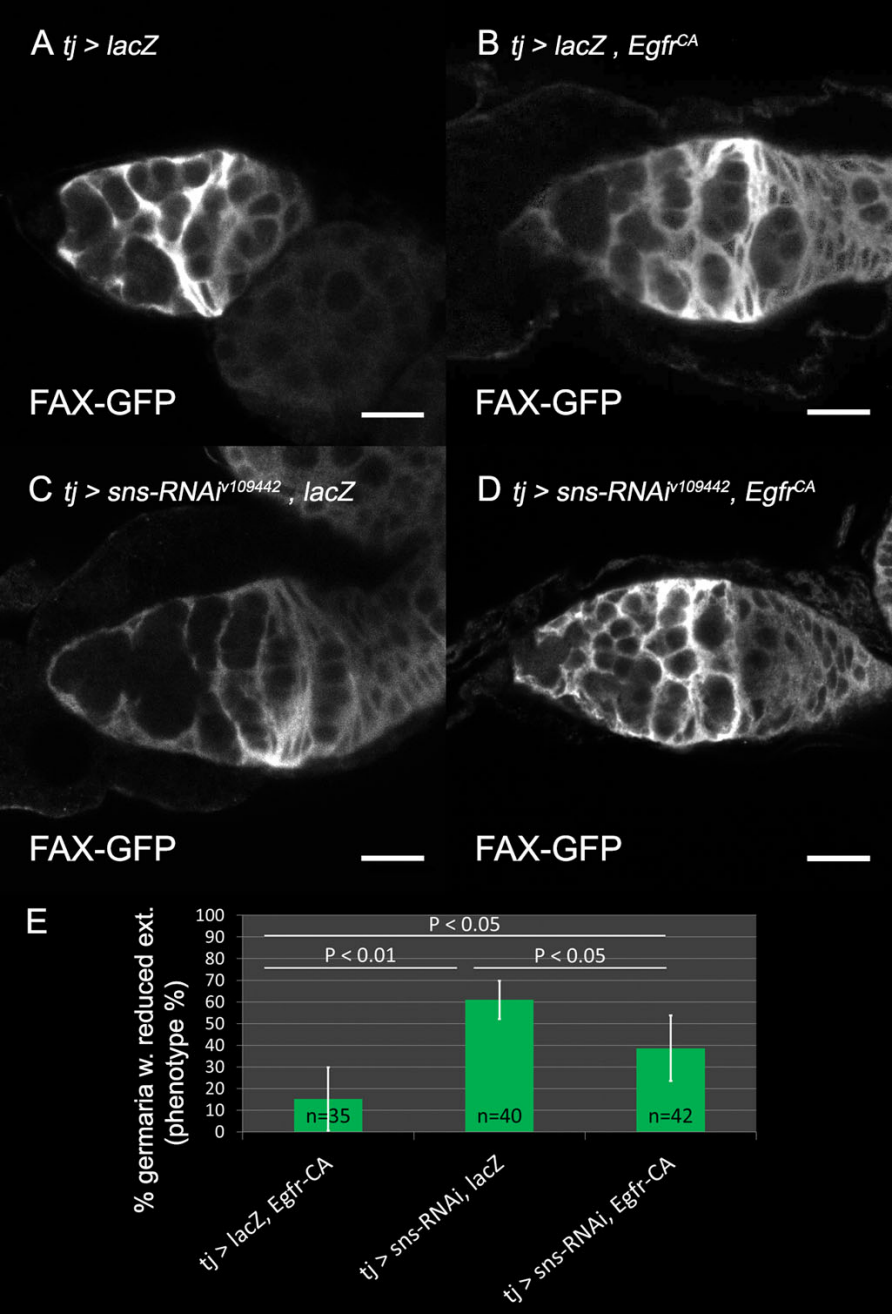
**Constitutive active Efgr in ECs partially rescues extension formation.** In all panels: FAX-GFP (white) marks somatic cell membranes. (A) FAX-GFP labels EC extensions. (B) Constitutively active Egfr (EGFR^CA^) in control germaria labeled with FAX-GFP and also carrying *UAS*-*lacZ*. (C) *sns* knockdown in the soma labeled with FAX-GFP and carrying *UAS*-*lacZ*. (D) *sns* knockdown in the soma combined with EGFR^CA^ in the soma labeled with FAX-GFP restores EC extensions. (E) Quantification of the percentage of germaria with reduced extensions in control (*n*=35), *sns* knockdown (*n*=40), or *sns* knockdown combined with constitutive active Egfr (*n*=42)*.* Four independent experiments are shown. *P*-values were calculated using binomial proportions z-test, bars represent s.d. Scale bars: 10 µm.

Next, a constitutively active Egfr (Egfr-CA) transgene was expressed in the ECs, in combination with an RNAi against *sns* to address its ability to rescue the extensions of the ECs in which *sns* was knocked down. We used *UAS*-*lacZ* to compensate for the addition of a UAS responsive site. Egfr-CA was indeed able to partially restore the formation of membrane extensions by the ECs. Whereas *sns-*RNAi caused decreased ECs extensions in 61% of the germaria (*n*=40), its co-expression with Egfr-CA decreased the reduction of EC extension to 38% of the germaria (*n*=42, *P*<0.05, Fig. 5, compare C to D, quantification in E). These results indicate that Egfr signaling in ECs can compensate for the loss of *sns*, and together they support the notion that IRM proteins act upstream of Egfr signaling in the ECs.

**Fig. 6.**
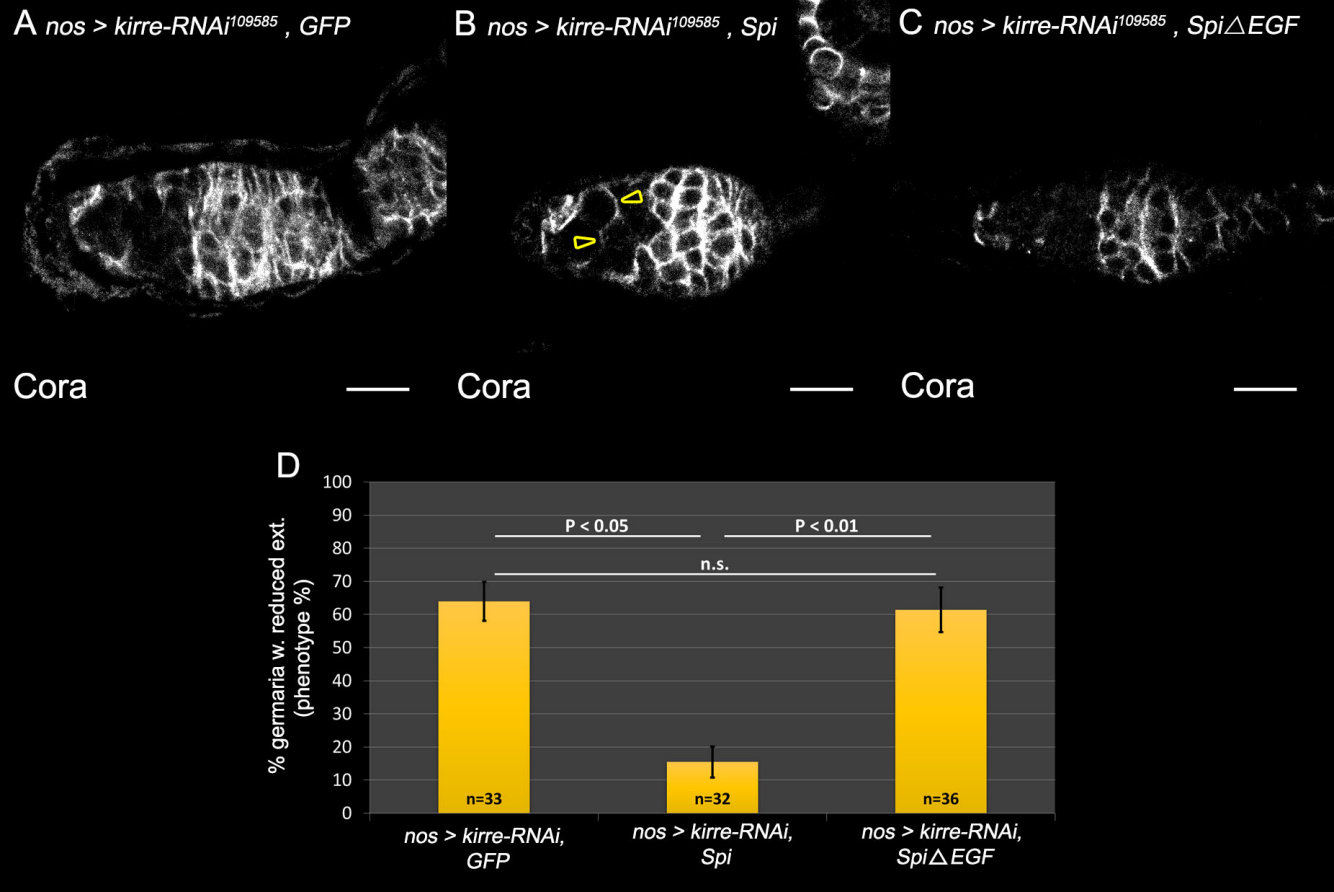
**Germline expression of Spi rescues EC extensions in *kirre* knockdown germaria.** In all panels: Anti-Coracle (Cora, white) marks EC membranes. (A) *kirre* knockdown in the germline results in reduced extensions. (B) Expression of Spi in the germline combined with *kirre* knockdown partially restores EC extensions (arrowheads). (C) Spi lacking the EGF domain does not restore extensions in the *kirre* knockdown background. (D) Quantification of EC extensions in *kirre* knockdown alone, in *kirre* knockdown expressed together with Spi (*n*=32) or *kirre* knockdown expressed together with inactive Spi (*n*=36), indicating a significant rescue by Spi. Three independent experiments are shown. *P*-values were calculated using binomial proportions z-test, bars represent s.d. Scale bars: 10 µm.

Previous reports support a model in which the Egfr ligand Spitz is provided by the GCs where it is cleaved and activated by the Rhomboid protease Stet (Gilboa and Lehmann, 2006; Liu et al., 2010; Schulz et al., 2002). Processing of Spi by Stet and its secretion from the GCs is necessary to activate the Egfr pathway non-autonomously in the ECs, leading to the formation of membrane extensions. In an attempt to further verify the hypothesis that IRM activity takes place upstream of Egfr signaling, we tested the ability of secreted active Spi driven in the GCs to rescue the phenotype of *kirre* knockdown in these cells. We used *UAS*-*GFP* to compensate for the addition of another UAS site. Significantly, membrane extensions of the ECs were restored in region 2a of the germaria in which *kirre* was knocked down by RNAi, relative to control in which *kirre* knockdown was combined with either *UAS*-GFP, or with *UAS*-Spi lacking the EGF domain. Quantification of these results showed that whereas 64% of the germaria exhibited reduced EC extensions following *kirre* knocked down alone (*n*=33), or in combination with inactive Spi (*n*=36), only 15% of kirre knocked down germaria which was combined with Spi showed reduced EC extensions (*n*=32, *P*<0.05, Fig. 6, compare B to A and C, quantification in D). We therefore conclude that Egfr signaling is acting downstream of the IRM cassette.

## DISCUSSION

**Fig. 7.**
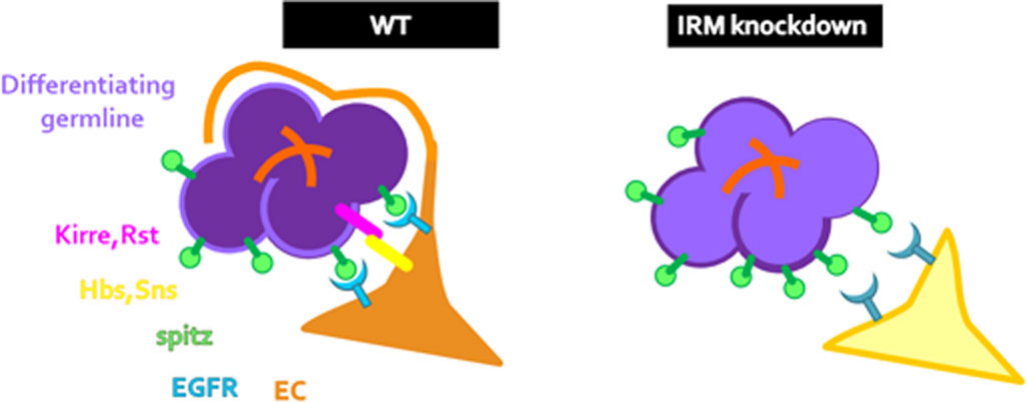
**Model: IRM maintains ECs and germline within signaling distance enabling EC extension formation.** In IRM knockdown, attachment of ECs and germline is hindered, increasing the distance between these two cell types. As a result, the soluble ligand Spi does not activate Egfr signaling in ECs to the same extent and less membrane protrusions form and encapsulate the germline.

A positive feedback loop between ECs and GCs ensures germline differentiation in the germarium. The ECs encapsulate the GCs with elongated membrane extensions, isolating them from signals originating from the maintenance niche, and promoting their differentiation, whereas the GCs further provide signals that promote the formation of these membrane projections by the ECs (Mottier-Pavie et al., 2016). Here we identified additional elements required for the reinforcement of EC-GC interaction, namely the four *Drosophila* members of the IRM protein family. These proteins function differentially in each of the cell types and presumably stabilize and shorten the distance between the two opposing plasma membranes. Our findings imply that *kirre* and *rst* are required exclusively in the germline, while *hbs* and *sns* function in the ECs. We suggest that stabilization of ECs membrane extensions and shortening the distance between the membranes of the two cell types are essential for efficient Egfr activation by the short range ligand Spi (Fig. 7).

Other systems in which IRM proteins function to maintain heterotypic interactions between cells had been described. In the *Drosophila* eye, similar pairs of IRM proteins are differentially expressed by ommatidial (Hbs and Sns) or inter-ommatidial (Kirre and Rst) cells and promote their preferential adhesion (Bao et al., 2010). Similarly, IRM proteins function to mediate proper spacing between bristles in the anterior D–V border of the wing imaginal disc (Linneweber et al., 2015). Whereas in these systems IRM proteins were not described to promote better signaling between the heterotypic cell types, such function cannot be excluded.

Full penetrance of the phenotype of the IRM could not be achieved since homozygous mutant suffer from defects in other tissues, and do not survive to adulthood. Furthermore, clonal analysis led to isolated mutant cells (either ECs or GCs) surrounded by WT cells, and since the IRM function non-autonomously the mutant cells were often rescued by their neighboring WT cells. When comparing the phenotype of EC membrane extensions between the heterozygous females and the knockdown cells, we noticed that the heterozygous mutant phenotype was higher relative to the tissue specific knockdown. This was unexpected and could possibly support a broader functional contribution of a given IRM, in both GCs as well as in ECs.

ECs must balance between two contradictory demands – the need to extend membrane protrusions between the GCs maintaining their tight encapsulation, and the need to release the germ cells allowing their progression through the germarium. IRM proteins mediate attachment, therefore must permit dissociation and re-attachment of the GCs to the ECs. Low levels of IRM constituents may control this grip-loosening behavior through a comparable number of attachment sites between the two cell types. Such low levels are consistent with the haploinsufficiency of the phenotype of EC membrane extensions in the IRM mutants. In such a model, maximal grip would depend on the number of EC membrane extensions, which express relatively low levels of IRM proteins. Loosening might take place when the number of extensions decreases by lowering Egfr signaling.

An interesting outcome from our experiments was that the degree of EC extension phenotype and the extra number of round fusomes did not always correlate. It is therefore suggestive that GC differentiation defects are apparently influenced by additional factors which are independent of EC extensions.

Spi is a diffusible paracrine ligand of the Egfr, which undergoes several phases of processing from a pro-protein inactive form into an active ligand, including, Spi processing, its export to the membrane, and its further palmitoylation at its N-terminal end (Miura et al., 2006). Spi palmitoylation retains its association with the plasma membrane, restricting its range of activity. Spi palmitoylation in the GCs might limits its range of activity only to proximal ECs. It is hypothesized that IRM proteins activity is possibly required in this case to close the gap between the two membranes, allowing efficient Egfr activation in the ECs.

In summary, our results identify novel essential components, the IRM proteins, which act to mediate heterotypic interactions between ECs and GCs, upstream of the Egfr signaling pathway, necessary for encapsulation of the GCs by ECs, and induce their further differentiation.

## MATERIALS AND METHODS

### Fly stocks

*tj*-Gal4 is a NP insertion (P{GawB}NP1624) into the *traffic jam* gene, and was obtained from the Drosophila Genetic Resource Centre. *nos*-Gal4 was from Dr Ruth Lehmann (NYU, USA). FAX-GFP originated in Yale FlyTrap (Quinones-Coello et al., 2007). *UAS-lacZ* was provided by Dr Jessica Treisman (NYU School of Medicine, USA). *sns^xb3^* (Bour et al., 2000), *sns^s660^* (Shelton et al., 2009) and hbs^459^ (Artero et al., 2001) were a gift from Dr Susan Abmayr (Stowers Institute for Medical Research). *Oregon Red* (BL#5), *rst[MI04842-GFSTF.1]/FM7j,B[1]* (BL#59410), *mFRP(nls), hs-flp FRT19A* (BL#31418) and *FRT19A* (BL#1744) were from Bloomington Stock Center*. UAS-EGFR^CA^*, *UAS-Spi*, *UAS-Spi*Δ*EGF* (Tsruya et al., 2002) and *Df67k30(duf, rst)/FM7,GFP* (Ruiz-Gómez et al., 2000) were a gift from Dr Benny Shilo. All RNAi lines used are detailed in Table S1 and in Table S2.

### Antibody staining

Antibodies were used in the following concentrations: from the Developmental Studies Hybridoma Bank (DSHB), mouse monoclonal anti-Hts (1B1, 1:20) deposited by Lipshitz, H.D. and mouse monoclonal anti-Coracle (1:200) deposited by Fehon, R.; guinea pig anti-traffic-jam (1:700) was from Dr Dorothea Godt (University of Toronto); rabbit anti-Diphosphorylated ERK (1:200, #4370) was from Cell Signaling; rabbit anti-GFP (1:1000, #ab290) was from Invitrogen. Rabbit anti-Zfh1 (1:5000) was a present from Dr Ruth Lehmann (NYU, USA). Mouse anti-Rst (1:25) was a gift and from Dr Ricardo Guelerman P. Ramos (University of São Paulo, Brazil) from Dr Renate Renkawitz-Pohl (Philipps-Universität Marburg). Secondary antibodies were from Jackson Immunoresearch or from Invitrogen. Fixation and immunostaining of young adult ovaries were performed according to standard protocols. Images were acquired on Zeiss LSM 710, on a Zeiss Observer.Z1 or on Zeiss LSM 800 confocal microscope with Zeiss C-Apo-chromat 40×/1.20-W Korr M27 lens.

### Quantification of dpERK and Zfh1 staining intensity

Control and experimental animals were dissected and stained on the same day. Images acquisition was on the same day, with the same parameters. The brightest section for each EC was measured with the measure tool in ImageJ software.

### Quantification of fusome and EC numbers

Confocal images with plane to plane distance of ∼2.5 μm were analyzed. Round fusomes were counted, dividing (‘exclamation mark’) fusomes were excluded from the count. Distinct ECs were counted manually in all planes excluding the top and bottom planes as to exclude follicle cells.

### Statistical analyses

Experiments were repeated as reported in text. Statistical analysis of extension phenotype was by Binomial proportions z-test (error for binary scoring of germaria), statistical analysis of round fusome amount and of EC numbers was by two-sample *t*-test. Round fusome count did not include elongated fusomes of dividing GSCs. Bars represent s.d.

## Supplementary Material

Supplementary information
