# Supplementary material for: Escort cell encapsulation of Drosophila germline cells is maintained by irre cell recognition module proteins
Source: Biol Open. 2019 Mar 15;8(3):bio039842. doi: 10.1242/bio.039842 (PMC6451344; doi:10.1242/bio.039842)
Supplement: Supplementary information [file biolopen-8-039842-s1.pdf]

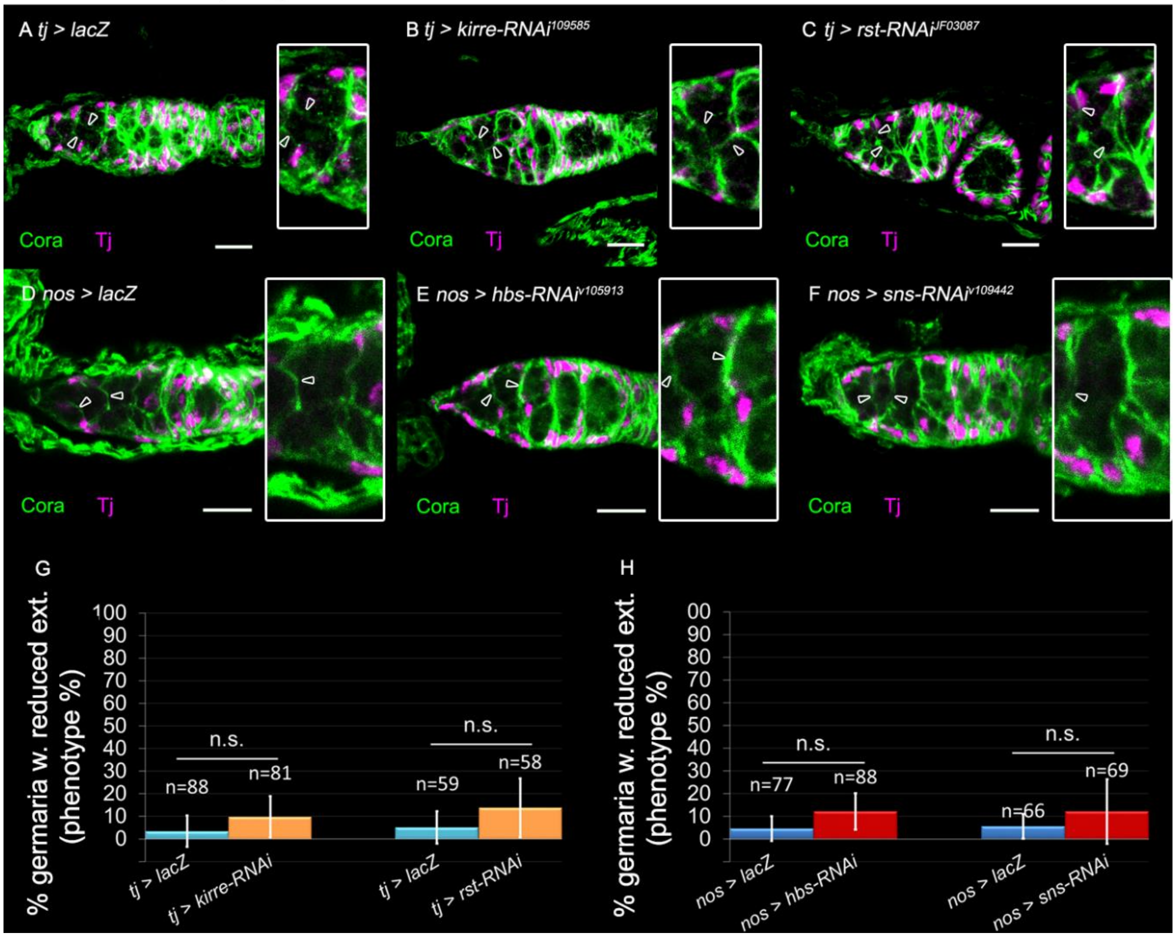

Figure S1. ECs membrane extensions are unperturbed by somatic knock down of *kirre* or *rst* and by germline knock down of *hbs* or *sns*

In all panels: anti-Coracle (Cora, Green) marks somatic cell membranes, anti-Tj marks ECs (Magenta). Arrowheads point to existing extensions, insets show region 2a magnified two-folds. (A) Control somatic *tj*-GAL4>lacZ germaria exhibit normal EC extensions. (B) Representative image of somatic knockdown of *kirre* (using *kirre* RNAi). (C) Representative image of somatic knockdown of *rst* (using *rst* RNAi). (D) Control, *nos*-GAL4>*lacZ* exhibits normal EC extensions. (E) Representative image of germline knockdown of *hbs* (using *hbs* RNAi). (F) Representative image of germline knockdown of *sns* (using *sns* RNAi). (G) Quantification of the percentage of germaria

with reduced extensions in somatic knockdown of *kirre* (n=81, seven independent experiments) or *rst* (n=58, six independent experiments). p-Values were calculated using binomial proportions z-test, bars represent S.D. (H) Quantification of the percentage of germaria with reduced extensions observed in germline knockdown of *hbs* (n=88, six independent experiments) or *sns* (n=69, five independent experiments). p-Values were calculated using binomial proportions z-test, bars represent S.D. Scale bar: 10µm.

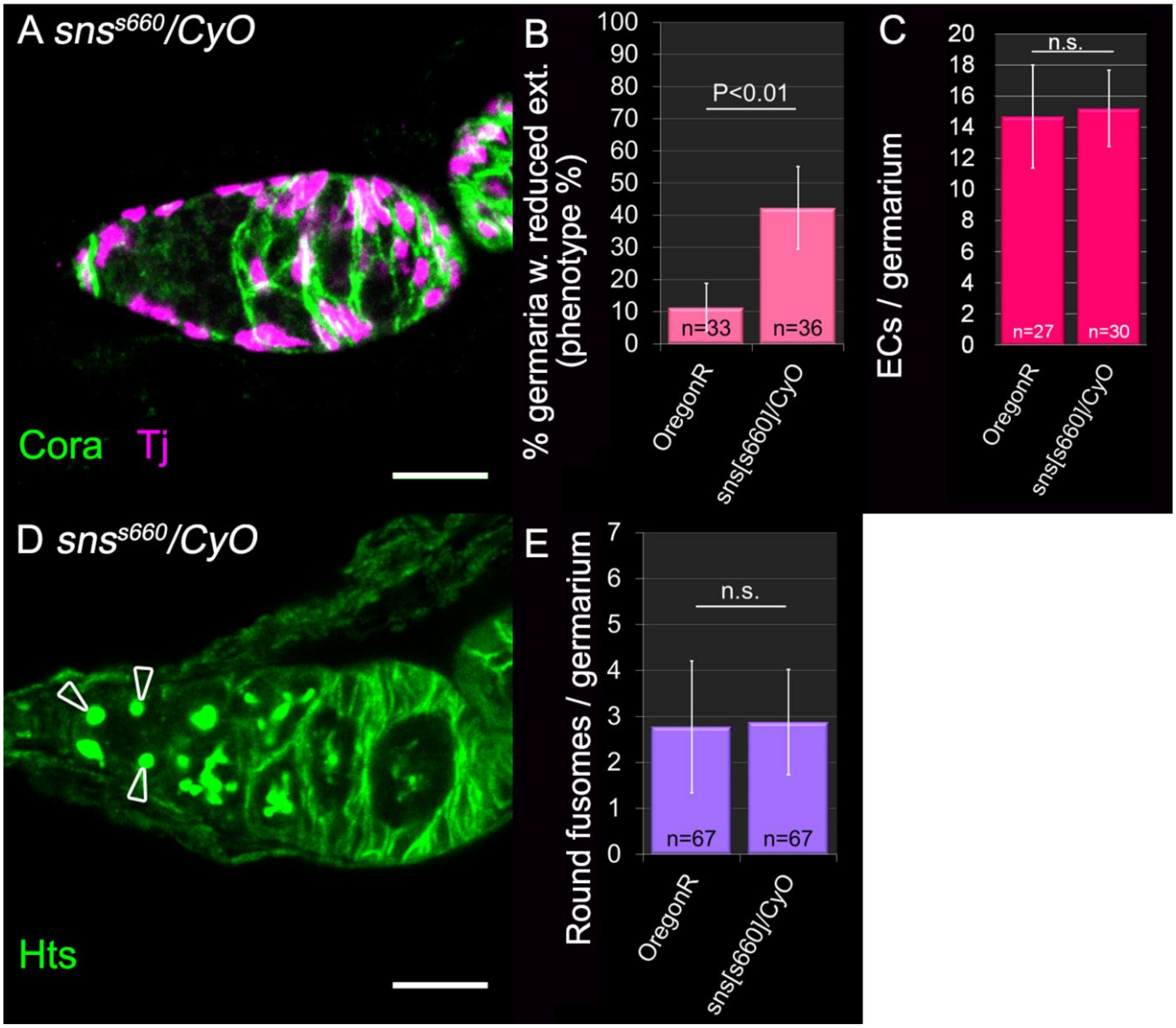

Figure S2. Phenotypic analysis of *sns*^{s660}

(A) Heterozygous mutants of *sns*^{s660} exhibit reduced membrane extensions (Cora, Green) of ECs (Tj, Magenta). (B) Quantification of the loss of membrane extensions in heterozygous *sns*^{s660} germaria (n=36, three independent experiments are shown). p-value was calculated using binomial proportions z-test, bars represent S.D. (C) Quantification of ECs present in heterozygous *sns*^{s660} germaria (n=30, three independent experiments are shown). p-Values were calculated using Two-Sample t-test, bars represent S.D. (D) Germline differentiation monitored by the number of round fusomes is unaffected in heterozygous mutants of *sns*^{s660}. (E) Quantification of the number of round fusomes in D. Six independent experiments are shown (n=67). p-Values were calculated using Two-Sample t-test, bars represent S.D. Scale bar: 10µm.

Biology Open • Supplementary information

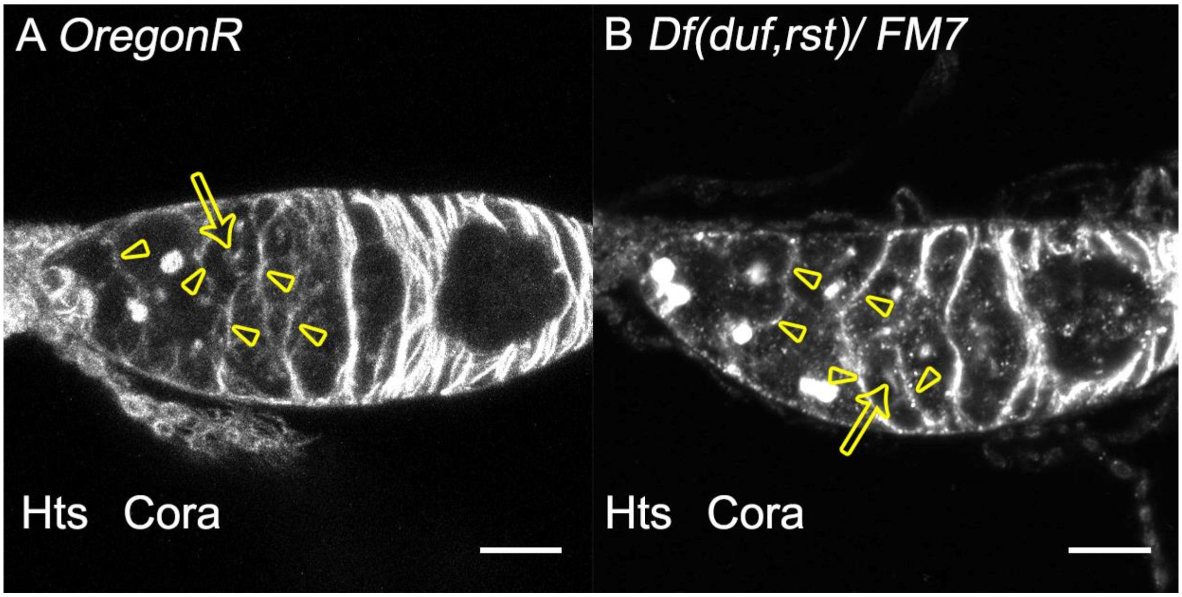

Figure S3. Phenotypic analysis of *kirre,rst* heterozygote

In all panels: anti-Hts (White) stains fusomes and anti-Cora (White) marks somatic cell membranes. Arrowheads point to EC extensions and arrows point to differentiating germline. (A) WT germaria have EC extensions and differentiating germline throughout. (B) Heterozygotes germaria for deficiency which deletes both *kirre* (*duf)* and *rst* exhibit normal EC extensions as well as differentiating germline. Scale bar: 10µm.

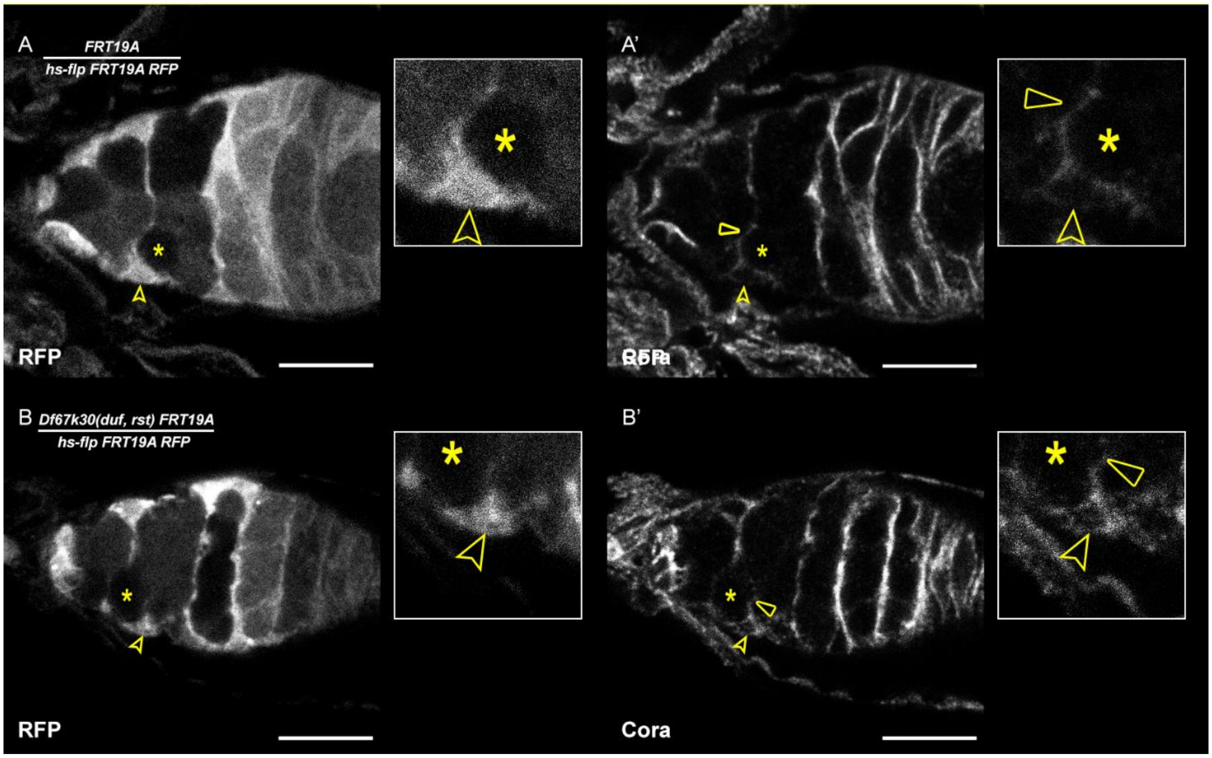

Figure S4. WT germline compensates for mutant GC clones

In all panels: Inset shows two-fold magnification, wedge points to an EC which touches a germline clone and a WT GC, the GC clone is marked by an asterisks. (A-A’) Control germaria. EC (wedge) touches a GC clone (asterisks, RFP negative) as well as a WT GC (RFP positive). (A’) The same EC (wedge) forms extensions (Cora, arrowhead). (B-B’) Heterozygotes germaria for deficiency which deletes both *kirre* (*duf)* and *rst*. (B) EC (wedge) touches a mutant clone GC (asterisks, RFP negative) as well as a heterozygous GC (RFP positive). (B’) This same EC (wedge) is able to form extensions (Cora, arrowhead). Scale bar: 10µm.

Biology Open • Supplementary information

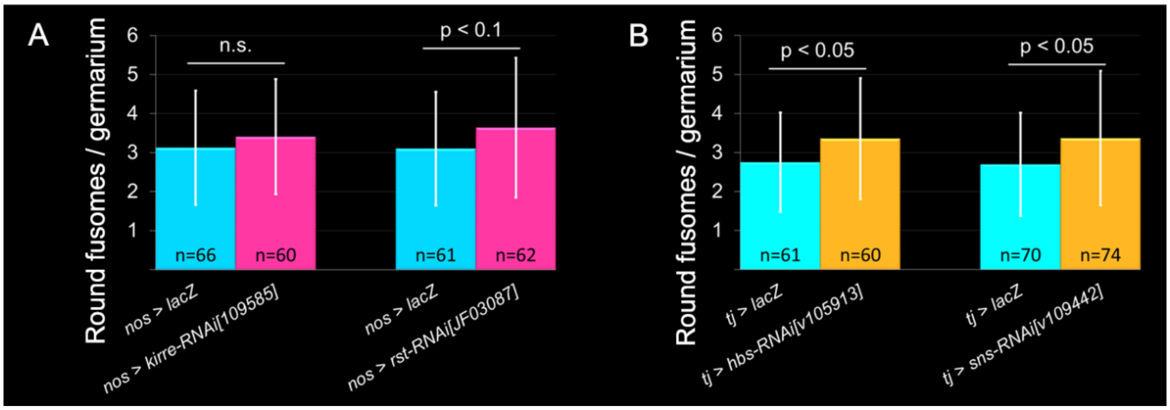

Figure S5. GC differentiation is hindered but not prevented in IRM knockdown

(A) Quantification of germline cells with round fusomes in germline knockdown of *kirre* (n=60) and *rst* (n=62) (five independent experiments are shown for each). (B) Quantification of germline cells with round fusomes in somatic knockdown of *hbs* (n=60, five independent experiments are shown) and *sns* (n=74, four independent experiments are shown)**.** p-Values were calculated using Two-Sample t-test, bars represent S.D.

Biology Open • Supplementary information

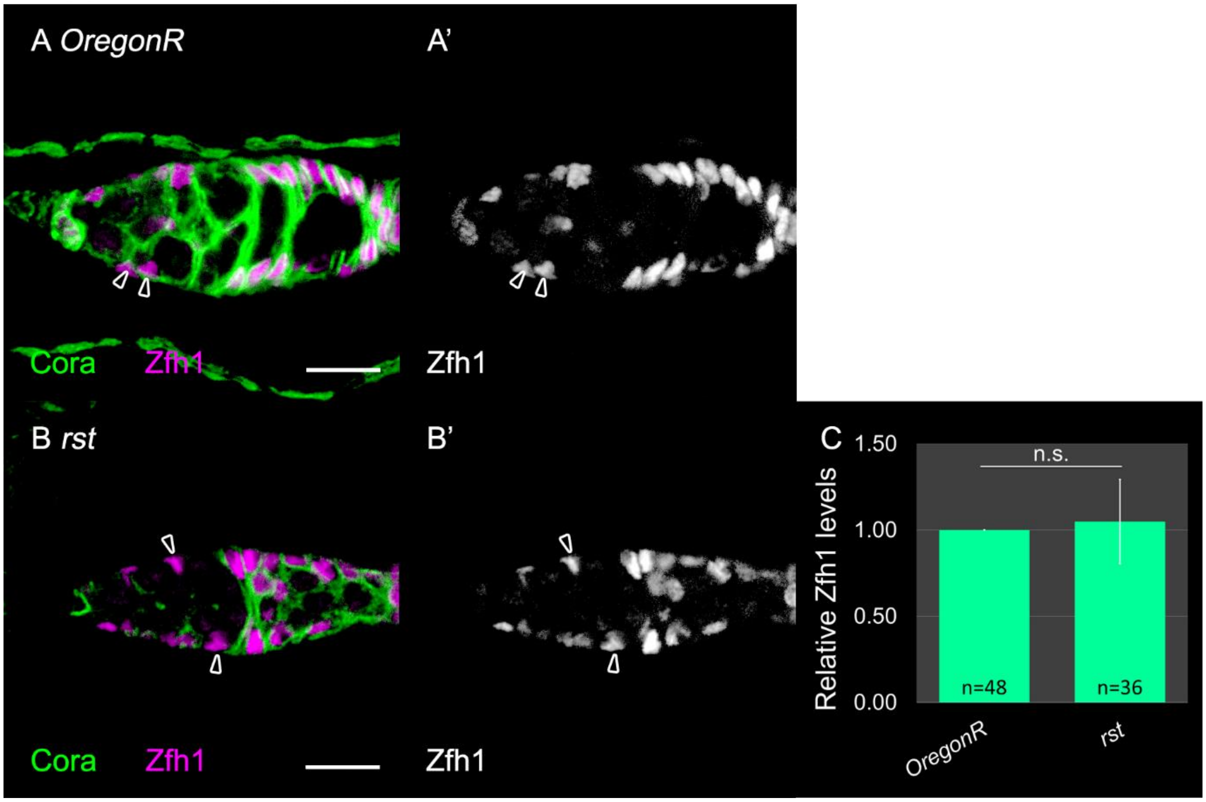

Figure S6. ***rst*** **is not required for STAT activation in ECs**

In all panels: anti-Coracle (Cora, Green) marks somatic cell membranes, anti-Zfh1 (Magenta) marks somatic cell bodies. (A-A') Control ECs express Zfh1. (B-B') *rst* homozygous mutant ECs have comparable Zfh1 labeling. (C) Quantification of Zfh1 fluorescent levels in ECs of control (OregonR, n=48) or *rst* (n=36) mutant germaria. Four independent experiments are shown. p-Values were calculated using Two-Sample t-test, bars represent S.D. Scale bar: 10µm.

Table S1. Genes identified in the screen

Genes and RNAi lines which elicit a phenotypic response in the different compartments tested. Plus and minus denominate presence or lack of phenotype, respectively.

Gene	Fly lines	*nos*-Gal4 (Germline)	*bab*-Gal4 (Maintenance niche)	*tj*-Gal4 (Escort Cells)
18 wheeler	*HM0524118*	+	-	+
AdamTS-A	*v33347, v110157*	-	+	+
BM-40-SPARC	*HMS02133*	-	+	+
cad86C	*v21327*	+	-	-
cad87A	*v105901*	+	-	+
cad99C	*v3739, v27212*	+	+	-
cad-N	*v1092, v101642*	-	+	+
cadN2	*v47538*	-	-	+
cals	*v105111*	-	+	-
CG13830	*HMC02924*	+	-	-
CG2247	*JF01328*	+	-	-
CG31619	*v33102*	+	-	-
CG31999	*JF01162*	+	-	-
CG4096	*v108353*	-	-	+
CG42709	*HM05068*	+	-	-
CG5003	*JF01510*	-	+	+

Biology Open • Supplementary information

CG5550	*v31001*	-	+	-
CG7800	*HM05133*	+	-	-
closca	*v104142*	+	-	-
collagen type IV	*HMC02910*	-	+	+
dachsous	*v4313*	-	-	+
eyes shut	*v22541*	-	-	+
fat	*v108863*	+	-	-
flightless I	*JF02720*	-	-	+
gfrl	*v103523*	+	-	-
inflated	*v44885, v100770*	+	-	+
kin of irre	*v109585, v27227, v6696*	+	-	-
kugelei	*v3749, v27114*	+	+	-
L(2)gl	*v51249*	-	+	-
lambik	*HM05114*	+	-	+
laminin A	*JF02908*	+	+	+
laminin B2	*v42559, v104013*	-	+	+
lcp1	*v30792*	-	-	+
lcp2	*v12537*	+	+	-
lcp3	*v107682*	-	+	-

Biology Open • Supplementary information

lapsyn	*HM05187*	+	-	-
lox2	*v33252*	+	-	-
mew	*v44890, v109608*	+	+	+
midline fasciclin	*v103621*	+	-	-
MMP1		-	+	+
m-spondin	*v15194, v107608*	+	-	+
muc12Ea	*v50435, v100177*	+	+	-
muc18B	*v100171*	+	-	-
muc26B	*v104205*	+	-	-
muc68D	*v25489, v105461*	+	-	-
muc91C	*v9867*	+	+	+
mur24F	*v7836, v44029*	-	-	+
mur29B	*v46704*	-	+	-
mur2B	*HMC02403*	-	-	+
mys	*HMS00043, JF02819*	+	+	+
netrin-B	*HMS01177*	+	-	-
nidogen/entactin	*v109625*	-	-	+
papilin	*v108005*	-	+	-

Biology Open • Supplementary information

pericardin	*v41320, v100357*	+	-	-
plod	*v45484*	-	+	+
polychaetoid	v38863, v104159	+	-	+
scribbled (scrib)	*HMS01490, HMS01993*	+	+	+
sda	*HMS01636*	-	+	+
shotgun	*v27082, v103962*	-	+	+
slit (sli)	*JF01228, JF01229*	+	-	+
trol	*JF03376, HMS01759, GL01153*	-	+	+
thrombospondin	*v7535*	+	-	-
tiggrin	*JF01143*	+	-	-
toll	*JF01276*	-	-	+
toll-9	*HMS00171*	-	-	+
tilB	*JF03324*	-	+	+
transglutaminase	*v103601*	-	+	-
twdlE	*v24867*	-	+	-
twdlG	*v100335*	-	-	+
twdlJ	*v103260*	-	-	+

Biology Open • Supplementary information

twdlT	*v107928*	-	-	+
U2A	*HMS00535*	+	+	+
viking	*HMC02400*	-	+	+
windpipe	*HM05118*	-	-	+

Biology Open • Supplementary information

Table S2. IRM RNAi lines

Fly line	Origin	Stock number
kirre-RNAi	VDRC	109585
rst-RNAi	Bloomington	TRiP.JF03087
hbs-RNAi	VDRC	105913
sns-RNAi	VDRC	109442

Biology Open • Supplementary information
